# Immunological Functions of the Membrane Proximal Region of MHC Class II Molecules

**DOI:** 10.12688/f1000research.7610.1

**Published:** 2016-03-17

**Authors:** Jonathan Harton, Lei Jin, Amy Hahn, Jim Drake

**Affiliations:** 1 Center for Immunology and Microbial Disease, Albany Medical College, Albany, New York, 12208-3479, USA; 2 Transplantation Immunology Laboratory, Albany Medical College, Albany, New York, 12208-3479, USA

**Keywords:** Myasthenia gravis, membrane proximal region, membrane distal region, immubiology, Class II connecting peptide

## Abstract

Major histocompatibility complex (MHC) class II molecules present exogenously derived antigen peptides to CD4 T cells, driving activation of naïve T cells and supporting CD4-driven immune functions. However, MHC class II molecules are not inert protein pedestals that simply bind and present peptides. These molecules also serve as multi-functional signaling molecules delivering activation, differentiation, or death signals (or a combination of these) to B cells, macrophages, as well as MHC class II-expressing T cells and tumor cells. Although multiple proteins are known to associate with MHC class II, interaction with STING (stimulator of interferon genes) and CD79 is essential for signaling. In addition, alternative transmembrane domain pairing between class II α and β chains influences association with membrane lipid sub-domains, impacting both signaling and antigen presentation. In contrast to the membrane-distal region of the class II molecule responsible for peptide binding and T-cell receptor engagement, the membrane-proximal region (composed of the connecting peptide, transmembrane domain, and cytoplasmic tail) mediates these “non-traditional” class II functions. Here, we review the literature on the function of the membrane-proximal region of the MHC class II molecule and discuss the impact of this aspect of class II immunobiology on immune regulation and human disease.

## Introduction

**Figure 1.**
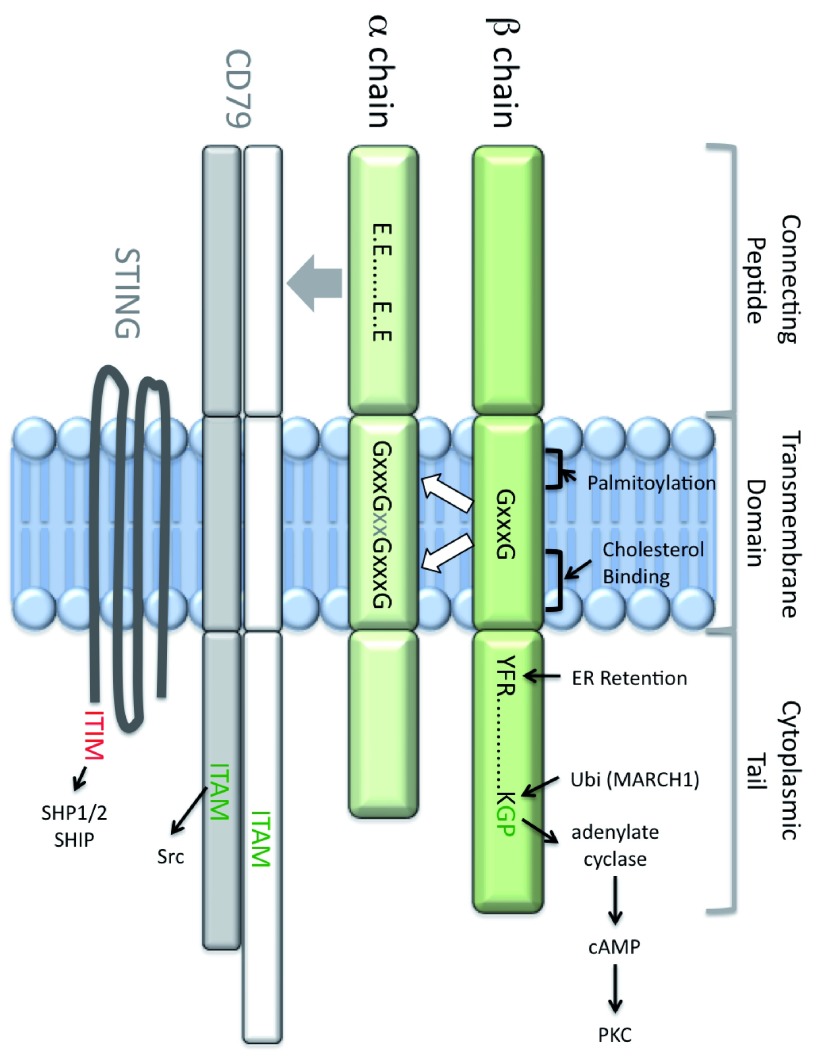
Major histocompatibility complex class II membrane-proximal region. The major histocompatibility complex class II membrane-proximal region (MPR green) is composed of the connecting peptide (CP), transmembrane (TM) domain, and cytoplasmic tail (CT). The α chain connecting peptide (αCP) controls class II association with both CD79 (which bears a cytoplasmic ITAM motif) and STING (which bears a cytoplasmic ITIM motif). CD79 association is dependent on an αCP motif, composed of four glutamic acid (E) residues. The class II TM is the site of palmitoylation and contains both GxxxG dimerization motifs and a cholesterol-binding motif. Together, these motifs control association of the α and β chain TM domains, which control lipid raft partitioning, CD79 association, and the structure and function of the class II extracellular domain. The class II β chain CT bears three known motifs: a membrane-proximal YFR motif that regulates activity of the invariant chain (Ii) endoplasmic reticulum (ER)-retention motif, a lysine (K) residue that is the target of MARCH1-mediated class II ubiquitination, and a membrane-distal GP motif that is necessary and sufficient to drive cyclic AMP (cAMP)-based class II signaling. ITAM, immunoreceptor tyrosine-based activation motif; ITIM, immunoreceptor tyrosine-based inhibitory motif; PKC, protein kinase C; STING, stimulator of interferon genes.

Major histocompatibility complex (MHC) class II molecules present antigen-derived peptides to T cells to drive immunological events such as thymic selection, activation of naïve CD4 T cells, and triggering of CD4 T-cell effector function. These events depend on T-cell receptor (TCR) recognition of peptide-class II complexes, the biochemistry and immunology of which have been the focus of much research. Both peptide binding and TCR engagement involve the membrane-distal region of the class II molecule. However, the class II membrane-proximal region (MPR), comprised of the extracellular connecting peptide (CP), transmembrane domain (TM), and intracellular cytoplasmic tail (CT), is not an inert base, merely supporting the molecule’s peptide binding/TCR-interacting region. The MPR ( Figure 1) drives multiple functions such as trafficking, signaling, and membrane partitioning, which are discussed below.

## Overview

As the study of class II immunobiology grew, both antigen presentation and other class II functions were reported. However, the desire to understand the role of class II in TCR engagement/T-cell activation led investigators to focus on peptide-class II complex generation and recognition, leaving the now non-traditional class II functions in relative obscurity. This review revisits some early findings as well as more recent discoveries that shed new light on these frequently overlooked but significant class II functions.

Class II immunobiology is more complex than simple presentation of peptides for TCR recognition. Class II molecules are multi-functional and have complex biological properties. For example, class II localization within lipid rafts and its association with tetraspan protein domains and cytoskeleton all impact the ability of antigen-presenting cells (APCs) to present antigen to T cells. Class II also drives intracellular signals activating tyrosine, serine/threonine, and inositol kinases through mediators, including intracellular calcium and cyclic AMP (cAMP). These pathways can lead to APC activation or death. In most cases, proteins such as class II-associated CD79 ^1, 2^ and STING ^3^ drive these events. Importantly, the class II MPR mediates essentially all of these functions.

This review provides an up-to-date analysis of the documented functions of the class II MPR, considers how human polymorphisms in the class II MPR might impact function (potentially providing insights into the molecular mechanism of disease), and highlights outstanding questions that should be the focus of future study.

## Human major histocompatibility complex class II polymorphisms

The human class II molecules HLA-DR, -DP, and -DQ have been linked to numerous diseases, including rheumatoid arthritis ^4– 6^, Goodpasture’s disease ^7^, multiple sclerosis ^8, 9^, narcolepsy ^10^, type I diabetes ^6^, Grave’s disease ^11^, celiac disease ^4^, and sarcoidosis/Lofgren’s syndrome ^12, 13^. Human leukocyte antigen (HLA) class II molecules also play a critical role in tissue/organ allograft success and susceptibility to infectious disease. Hence, an understanding of HLA immunobiology is essential to understanding the role of HLA in human health.

Much of the work to understand mechanisms linking class II to human health has focused on disease-associated polymorphisms in and around the membrane-distal peptide/TCR-binding region of the molecule. This approach has yielded a large body of information. For example, the principal HLA association for celiac disease is the heterodimer DQA1*05:01, DQB1*02:01 with a secondary association of DQA1*03, DQB1*03:02, encoded in cis or trans ^14^. These heterodimers present wheat gluten gliadin peptides modified by tissue transglutaminase 2 (released from damaged intestinal tissue), which converts glutamine residues to glutamic acid. Presentation of the modified peptides by the disease-associated class II leads to CD4 T-cell responses, cytokine production, additional tissue damage, and upregulation of HLA-DQ, causing amplification of the response ^15^. However, mechanisms underlying other disease linkages are less clear, and the MPR may hold some important clues.

Contrary to some statements in the literature, HLA class II polymorphisms are *not* restricted to the membrane-distal region of the molecule. Even though the sequence of the class II MPR is under-studied/under-reported (see below), analysis of existing databases illustrates the high level of MPR polymorphism ( Table 1). Moreover, many of these polymorphisms lie in or near functional domains (discussed in detail below), suggesting that they impact class II structure or function (or both) and thus human health.

**Table 1.** Amino Acid Sequence Comparison of Murine and Human Class II Molecules ^1^.

Chain	Connecting peptide – – Transmembrane – – Cytoplasmic
I-A α	M1 M2 WEPEIPAPMSELTET VVCAL GLSVG LV GI V VG TI F II QGLRSGGTSRHPGPL
I-E α	WEPEIPAPMSELTET VVCAL GLSVG LV GIVVG TIFII QGLRSGGTSRHPGPL
DRA*01:01:01:01	WEFDAPSPLPETTEN VVCAL GLTVG LV GIIIG TIFII KGVRKSNAAERRGPL
DRA polymorphisms	............... ...................... ...L...........
DPA1*01:03:01:01	WEAQEPIQMPETTET VLCAL GLVLG LV GIIVG TVLII KSLRSGHDPRAQGTL
DQA1*01:01:01 ^2^	WEPEIPAPMSELTET VVCAL GLSVG LV GIVVG TVFII QGLRSVGASRHQGPL
DQA polymorphisms ^2^	....N.T......G. ...T.....S.MC...D..L.. R..............
	
I-A β	AQSESARSKM LSGIG GCVLG VI F LG L GL F I RHRSQKG̲P̲RGPPPAGLLQ
I-E β	AQSTSAQNKML SGVG GFVLG LLFLGAGLFIYF RNQKG̲Q̲SGLQPTGLLS
DRB1*01:01:01	ARSESAQSKM LSGVG GFVLG LLFLGAGLFIYF RNQKGHSGLQP**T**GFLS
DRB polymorphisms	......Q....R... ...I...M.......T...... K........H.R.L..
DPB1*01:01:01	AQSDSAQSKT LTGAG GFVLG LIICGVGIFM HRRSKKVQRGSA
DQB1*05:03:01 ^2^	AQSESAQSKM LSGVG GFVLG LIFLGLGLII RQRSRKG̲P̲QGPPPAGLLH
DQB polymorphisms ^2^	.....DE..C..C.I ...IVCLG....IHA..V.N GH.NQ..∆∆∆∆∆∆∆∆...

1. **Motifs:** Connecting Peptide, CD79 association heavy underline (I-A only); Transmembrane Domain, GxxxG motifs indicated by dark grey box, putative cholesterol binding motif indicated by light grey boxes (I-A only); C targets of palmitoylation (not highlighted); Cytoplasmic Domain, G̲P̲ – cAMP motif double underlined, K – ubiquitination sites. DRB – CT GHS motif mediates cytoskeletal interaction (not indicated)

2. Missense polymorphisms compiled from the IMGT/HLA ( http://www.ebi.ac.uk/ipd/imgt/hla/) and SNP ( ensembl.org) databases.

## Class II cytoplasmic tail

The class II CT controls multiple aspects of class II immunobiology such as endoplasmic reticulum (ER) retention of class II-invariant chain (Ii) complexes and MARCH-mediated class II ubiquitination, which controls trafficking within the antigen-processing pathway. Together, the CT and TM domains control cytoskeletal association, which impacts T-cell activation. Finally, the CT is essential for certain class II-mediated signals, of which the cAMP pathway is the most studied.

In the ER, class II αβ dimers assemble on Ii trimers to form nonameric complexes that can exit the ER and traffic to peptide-loading compartments. Ii is a type II membrane protein with an N-terminal CT. Studies by Thibodeau and colleagues revealed that the human Ii p35 and p45 isoforms have an N-terminal extension (due to alternative upstream start sites) possessing an ER-retention motif, which allows ER retention of incomplete class II-Ii complexes ^16, 17^. They also demonstrated that the β chain CT of all three human class II molecules can mask the retention motif to allow egress of assembled complexes ^16, 17^. Interestingly, masking requires only the three β chain membrane-proximal CT residues (i.e. YFR in HLA-DR; Figure 1), suggesting that simple steric hindrance of the retention motif is *not* the mechanism of masking. Thus, the CT has an important role in controlling the early steps of class II biosynthesis.

Subsequent to Ii dissociation, class II molecules bind antigen-derived peptide and are delivered to the plasma membrane, from which they can cycle through the endocytic pathway. Roche and colleagues have shown that, within early endosomes, the ubiquitin ligase MARCH1 can ubiquitinate the class II CT ^18, 19^, which causes class II to be shunted out of the recycling pathway and into deep endocytic compartments for degradation ^20, 21^. Although there are reports of other ubiquitin ligases such as MARCH8 and MARCH9 ubiquitinating class II ^22, 23^, MARCH1 appears to be the main class II ubiquitin ligase in dendritic cells, B cells, and macrophages ^20, 21^.

MARCH1 targets the single conserved β chain CT lysine residue. Although arginine substitution of this lysine prevents class II ubiquitination, it is unclear whether and how flanking residues or the α chain CT impacts MARCH-mediated class II ubiquitination. A study by Thibodeau and colleagues revealed that the MARCH1 TM domain is critical for class II interaction/ubiquitination ^24^, suggesting a role for the class II TM domain in controlling class II ubiquitination. Consistent with this idea, Kelly and colleagues reported a role for the class II TM domain in HLA-DR interactions with the MARCH1 homologue MARCH8 ^22^. However, some of these interactions were defined with a chimeric molecule bearing an unpaired β chain TM domain, which may behave differently than the class II heterodimer (see below). Nevertheless, a picture is emerging where the class II CT and TM domains control both ER egress of class II-Ii complexes and ubiquitin-dependent delivery of class II to late endocytic compartments, influencing class II trafficking throughout the antigen-processing pathway.

Cell surface peptide-class II complexes interact with the APC cytoskeleton, impacting the ability of the APC to drive T-cell activation ^25– 28^. Using anti-class II antibodies, multiple labs have demonstrated the existence of class II-cytoskeleton interactions. It should be noted that many studies have employed somewhat ambiguous definitions of “cytoskeleton” (e.g., detergent-insoluble material) and have not defined the underlying molecular mechanisms. Nevertheless, in these studies, deletion of either class II α or β chain CT alone fails to completely sever cytoskeletal association, suggesting that both class II CTs are involved. Similarly, a substantial increase in class II lateral translation within the plasma membrane of the cell is seen only when both CTs are deleted ^29, 30^. In one study of human class II, Mourad and colleagues ^26^ demonstrate that, in the absence of an HLA-DR α CT, the DR β chain CT mediates cytoskeletal interaction and that this interaction is mediated at least in part by the β chain CT GHS motif ( Table 1).

Engagement of class II molecules leads to activation of multiple downstream signaling pathways. In many cases, class II-associated “accessory molecules” such as CD79 mediate signaling pathway activation (see below). However, in B cells, class II-driven intracellular cAMP signaling is directly dependent on the class II β chain CT ^31, 32^. Class II-driven cAMP signaling (or the addition of dibutyryl-cAMP) both enhances B-cell receptor (BCR)-mediated antigen processing ^33, 34^ and drives plasma cell differentiation ^35^. Thus, signals emanating directly from the class II CT synergize with signals emanating from other class II-associated signaling molecules to drive efficient APC activation.

A β chain CT GP or GQ motif ( Table 1) is necessary and sufficient for class II-driven cAMP production and protein kinase C (PKC) activation ^32, 36, 37^. This GP motif is present in I-A and HLA-DQ, while I-E has a comparably active GQ motif ^32^. The GH motif of HLA-DR bears some similarity to GQ and GP, and CT truncation mutants lacking the GH motif fail to activate PKC ^38^, but whether the specific GH motif is active is untested. In contrast, the VQ in HLA-DP is predicted to be incapable of driving a cAMP response based on loss-of-function GA mutants ^32^. The GP motif most likely couples class II to an undefined adenylate cyclase responsible for cAMP production and activation of downstream signaling molecules such as PKC.

Interestingly, the close proximity of the class II β chain CT ubiquitination, cytoskeletal, and cAMP signaling motifs ( Table 1) suggests that at a molecular level these functions may be mutually exclusive. For example, individual class II molecules that have undergone MARCH1-mediated ubiquitination (which attaches a large ubiquitin molecule to the small CT) may sterically block the adenylate cyclase interactions needed to drive cAMP signaling. This would mean that subsets of class II molecules (possibly bearing different sets of peptides such as self versus foreign antigen) could be biased toward particular CT-dependent functions such as cAMP signaling versus MARCH1-dependent ubiquitination. This would allow an APC to tailor both the expression of various peptide-class II complexes for TCR engagement and the APC’s response to TCR engagement of these complexes. This extent of regulation would provide a previously unappreciated level of sophistication to regulation of peptide-class II expression and signaling.

### Open questions

• What mechanism couples class II engagement to cAMP production? Which adenylate cyclase is responsible? What signaling pathways and *in vivo* immunological functions are cAMP-dependent?
• How is class II ubiquitination controlled? Is there a MARCH1 recognition motif? What are the immunological roles of class II ubiquitination?
• How does the class II β chain CT control activity of the Ii ER retention motif?
• What level of crosstalk is there between the CT ubiquitination, cAMP signaling, and cytosleletal interaction motifs?

## Class II transmembrane domain

The class II TM domain controls membrane domain partitioning and class II structure, both of which influence antigen presentation and T-cell activation. The TM domain also controls class II association with the CD79 signaling complex (see below) and with MARCH family ubiquitin ligases (see above), which regulate class II signaling and expression. Class II molecules also associate with tetraspan family member proteins such as CD82, which can form a web of interacting tetraspan proteins ^39– 41^. Although it is likely that these associations are driven by interactions between the TM domains of class II and the tetraspan proteins, the precise role of the class II TM domains versus other regions of the class II molecule such as the CT and CP is currently unclear (see discussion of class II-STING interactions below).

Many membrane proteins undergo fatty acylation (e.g., palmitoylation) of TM domain cysteine residues, which can control association with membrane domains such as lipid rafts. The TM domain of all class II α chains contains a highly conserved cysteine residue, as does the TM domain of the I-A and HLA-DP β chain ( Table 1). These conserved cysteine residues represent putative palmitoylation sites, and both I-A and HLA-DR have been shown to incorporate palmitic acid ^42, 43^. Moreover, mutation of I-A TM domain cysteine residues has been shown to decrease class II lipid raft partitioning and, when transduced into thymic epithelial cells, decreased class II-driven positive selection of CD4 ^+^ T cells ^43^. However, the molecular mechanism of class II palmitoylation and how it is controlled are currently not known.

Cosson and Bonifacino first demonstrated the impact of the class II TM domain on *extracellular domain* structure and function ^44^. They noted the unique enrichment/positioning of multiple TM domain glycine residues and demonstrated that mutation of these conserved residues results in an I-A ^k^ class II molecule that is recognized by a conformation-insensitive monoclonal antibody (mAb) (10-2.16) but *not* by a conformation-specific mAb (11-5.2). Subsequent work by King and Dixon revealed that the TM domain glycine residues form GxxxG dimerization motifs, which function by one-to-one pairing to facilitate TM domain interactions ^45^. The authors noted that while the class II β chain contains one GxxxG motif, the α chain contains two (an N-terminal M1 motif and a C-terminal M2 motif), and, using both *in silico* and *in vitro* studies, demonstrated that the HLA-DR β chain motif is able to pair with either α chain motif, albeit with differing “affinity” ^45^. Taken together, these studies revealed previously unknown dimerization motifs within the class II TM domain and suggest a critical role in class II structure/function.

Further studies established that the 11-5.2 anti-I-A ^k^ mAb is unique in that it selectively binds to *M1 paired* I-A ^k^ class II molecules, and that these molecules are enriched in plasma membrane lipid rafts ^46^ and have unique signaling properties ^47^. Although other anti-class II antibodies *may* discriminate conformers on the basis of alternative pairing of TM domain GxxxG dimerization motifs, we are unaware of any that have been identified and characterized. Interestingly, although M1 paired class II represents approximately 10% of cell surface I-A ^k^ molecules ^46^, they carry up to 100% of class II *immunological function*. Specifically, the 11-5.2 mAb blocks over 90% of antigen-specific B cell-T cell interactions *in vitro* ^46^ and *in vivo* T-cell activation ^48^. These findings are compatible with the results of Roche and colleagues, who demonstrated that lipid raft-resident peptide-class II (such as M1 paired class II) is more efficient at activating CD4 T cells, in part due to the clustering of the raft-resident complexes ^49^.

Formation of M1 paired class II is invariant chain (Ii)-dependent, as the addition of a class II tethered peptide that blocks Ii binding also blocks their formation ^46^. Because previous studies established that the Ii TM domain can interact with the class II TM domain ^50^, these results suggest that Ii may guide the pairing of class II TM domains, which would be consistent with the presence of M1 paired class II in the ER of the cell ^51^. Interestingly, GxxxG dimerization motifs are also present in the DM and DO molecules that control class II peptide loading ( Table 2), suggesting that this type of TM domain interaction may have additional functions within the class II antigen-processing pathway.

**Table 2.** Major histocompatibility complex class II transmembrane domain GxxxG motifs.

Chain	TM Domain Sequence
I-Aα	VVCAL GLSV GLV GIVV GTIFIIQGL
DMα	VLC GVAF GLGVL GIIV GIVLIIYF
H-2Mα	ALC GVAF GLGVL GTII GIVFFLCS
DOα	LVCAL GLAI GLV GFLV GTVLII
H-2Oα	LICGL GLVL GLM GCLL GTVLMI
	
I-Aβ	MLSGIG GCVL GVIFLGLGLFI
DMβ	VSVSAVTLGL GLIIFSLGVISW
H-2Mβ	VSVSAATLGL GFIIFCVGFFRW
DOβ	MLSGIAAFLL GLIFLLVGIVIQL
H-2Oβ	ILSGAAVFLL GLIVFLVGVVIHL

Human Sequences from “ http://www.ebi.ac.uk/imgt/hla/align.html”

In additional studies, Roy and colleagues have shown that membrane cholesterol, which is important for lipid raft structure, can affect expression of 11-5.2-reactive M1 paired I-A ^k^ class II molecules ^52^. The authors used a combined *in situ* and *in silico* approach to identify a putative TM domain cholesterol binding motif and demonstrated that mutation of this motif blocks both formation of M1 paired class II and the ability of cells to effectively present antigen to CD4 T cells.

**Figure 2.**
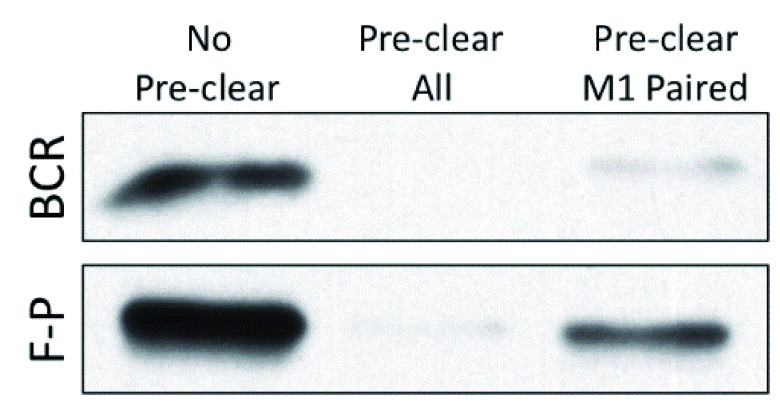
Differential peptide loading of M1 versus M2 paired major histocompatibility complex class II molecules. B cells were pulsed with hen egg lysozyme (HEL) antigen under conditions that lead to expression of similar levels of C4H3-reactive HEL _46-61_–I-A ^k^ peptide-class II complexes ^53^. For B-cell receptor-mediated processing (BCR), MD4.B10.Br B cells (expressing a transgenic HEL-specific BCR) were pulsed with 100 nM HEL protein. For fluid-phase processing (F-P), B10.Br B cells were pulsed with 100 µM HEL protein. As previously reported ^1^, cells were lysed and pre-cleared with protein G-Sepharose (PGS) only (No Pre-clear), 10-3.6 + PGS (Pre-clear all), or 11-5.2 + PGS (Pre-clear M1 Paired). Remaining (non-pre-cleared) HEL _46-61_–I-A ^k^ complexes were immunoprecipitated with the C4H3 monoclonal antibody. The amount of major histocompatibility complex class II β chain remaining in each sample was determined by Western blot ^1^. Although the 11-5.2 anti-M1 paired class II monoclonal antibody pre-cleared essentially all of the BCR-generated peptide-class II complexes, it pre-cleared only a fraction of complexes generated by fluid-phase antigen processing. Shown are representative results from one of three independent experiments.

Most recently, we have shown that M1 paired class II uniquely binds intracellular antigen-BCR complexes in a putative MHC class II peptide-loading complex (PLC) ^1^. In the PLC, M1 paired class II appears to acquire both peptide derived from the processing of BCR-bound antigen and a CD79 signaling module ^1, 2^. In contrast, peptide derived from fluid-phase processing of non-cognate antigen is loaded onto *both* M1 and M2 paired class II ( Figure 2). This finding is consistent with the observation that the B-cell response to engagement of peptide-class II complexes formed via BCR-mediated versus fluid-phase antigen processing is different ^53^, and could explain why *in vivo* B cell-T cell interactions subsequent to BCR-mediated antigen processing are prolonged and highly dynamic, whereas interactions in the absence of cognate antigen (presumably mediated by self-peptide loaded onto M1 and M2 paired class II) are transient ^54^.

Taken together, these studies reveal that TM domain pairing is controlled by both GxxxG dimerization motifs and a cholesterol-binding motif, impacting extracellular domain structure and the binding of the 11-5.2 mAb, which binds an epitope near the class II peptide-binding groove/TCR contact site ^46, 55^. TM domain pairing also controls both entry into the MHC class II PLC and class II lipid raft partitioning. Hence, the TM domain has profound effects on both the structure and immune function of class II molecules.

### Open questions

• How is class II palmitoylation and GxxxG motif pairing controlled?
• Is there crosstalk between TM domain palmitoylation, GxxxG motif pairing, and cholesterol binding?
• How do changes in the orientation of TM domain pairing impact the structure or function (or both) of the extracellular domain of the molecule (TCR binding, peptide binding, DM binding, CD4 binding, and CD79 binding; see below)?
• Does GxxxG motif pairing impact the functions of HLA-DM or HLA-DO?

## Class II connecting peptide

The class II extracellular domain is tethered to the cell surface via α and β chain CPs, which are 15 and 10 amino acids long, respectively ( Table 1). In addition to linking the extracellular and TM domains, CPs control class II interactions with signaling molecules. Studies on the function of the class II CP have focused on B cells, but some of the observations such as control of STING-mediated cell death are likely transposable to other APCs such as dendritic cells and macrophages.

In a recent study of the class II CP, Jin and colleagues established that in B cells the α chain CP (αCP) mediates class II interaction with CD79 (typically regarded solely as the BCR signaling subunit) and a then-uncharacterized molecule critical for class II-driven B-cell death (later determined to be STING, also known as MPYS, which is an integral membrane protein with four TM domains but *not* a member of the tetraspan family of proteins ^3^) ^56^. The authors also discovered that mutation of the four αCP glutamic acid residues causes decreases in CD79 association and downstream signaling pathways. This result is interesting as it reveals that extracellular domains control class II-CD79 interactions, distinct from CD79-BCR associations that are mediated instead by TM domain interactions. Subsequent work revealed that CD79 preferentially associates with M1 paired class II (see above) ^1^, suggesting that both the class II CP and TM domain may affect class II-CD79 association. Here, the impact of the class II TM domain could be either direct (the class II TM domain could interact with the CD79 TM domain) or indirect (class II TM domain pairing could affect the availability of αCP to interact with the CD79 extracellular domain).

The reported signaling of class II via CD79 ^2, 56^, as well as the reported association of class II with intact antigen-BCR complexes in a putative MHC class II peptide loading complex (PLC, 1), raises questions about the form of class II-associated CD79. Is the class II-associated CD79 just the CD79a/CD79b heterodimer, or is it part of an intact BCR complex? In the original report by Lang and colleagues ^2^, the authors demonstrate that class II engagement leads to selective phosphorylation of class II-associated CD79a but that membrane immunoglobulin engagement leads to selective phosphorylation of membrane immunoglobulin-associated CD79a, suggesting that class II is associated with a distinct pool of CD79 *not* associated with membrane immunoglobulin. This would be consistent with our finding that induction of BCR endocytosis *fails* to result in downregulation of cell surface class II molecules (suggesting that membrane immunoglobulin and class II are *not* physically associated at the cell surface; Drake, unpublished data). Moreover, these findings are consistent with the scenario suggested above, where complexes of intact cell surface BCR molecules (i.e. membrane IgH/L plus CD79) and bound cognate antigen are internalized and trafficked to the intracellular PLC, where BCR-bound antigen is converted to class II-bound peptide and the BCR CD79 signaling subunit is transferred to the newly formed peptide-class II complex. The ability of class II engagement to drive intracellular calcium signaling in resting B cells under some conditions ^47^ would suggest that constitutively internalized BCR molecules may also enter this pathway, generating some “baseline” level of class II-CD79 complexes even on resting cells.

STING has recently received attention as an innate immune receptor for cyclic dinucleotides. However, STING is also a class II-associated homodimer that possesses a cytoplasmic immunoreceptor tyrosine-based inhibitory motif (ITIM) ^3^, which can recruit the tyrosine and inositol phosphatases SHP-1 and SHIP. Hence, class II signaling is somewhat similar to BCR signaling in that engagement can elicit immunoreceptor tyrosine-based activation motif (ITAM) or ITIM signaling (or both) depending on conditions. Antigen engagement of the BCR results in CD79-driven ITAM signaling, whereas co-engagement of the BCR and ITIM-bearing FcγRII by immune complexes results in mixed ITAM/ITIM signaling. For class II, selective engagement of CD79-associated class II, such as with the M1 conformer-specific 11-5.2 mAb, results in robust BCR-like ITAM signaling. In contrast, ligation of *all* class II molecules with the pan-reactive 10-3.6 mAb fails to elicit a detectable intracellular calcium flux ^47^, likely because of recruitment of ITIM-bearing STING-associated class II molecules. Consistent with this scenario, ectopic overexpression of STING blocks CD79-mediated class II-driven intracellular calcium signaling ^3^.

These findings suggest that the relative levels of CD79 and STING associated with any particular MHC class II-peptide complex may be variable. Therefore, TCR engagement of peptide-class II complexes formed under different conditions or via different pathways (e.g., fluid-phase versus BCR-mediated antigen processing) may have profoundly different effects on the APC. TCR engagement of peptide-class II complexes having a high ratio of CD79 to STING would lead to predominantly ITAM-based class II signaling and APC activation, whereas TCR engagement of peptide-class II complexes having a lower ratio of CD79 to STING would result in predominantly ITIM-based class II signaling and APC death. This highlights the profound impact that functions mediated by the MPR of the class II molecule could have on the outcome of APC-T cell interaction.

### Open questions

• Is binding of CD79 and STING to class II a mutually exclusive event?
• Do all class II molecules associate with CD79 or STING or both? What controls class II association with CD79 and STING?
• What are the immunological functions of peptide-class II complexes associated with CD79, STING or both molecules?

## Human class II membrane-proximal region and disease

Current sequence-based protocols for clinical typing of HLA class II molecules focus on the extracellular domain of the molecule, extending from the leader peptide to the C-terminal end of the immunoglobulin domain, *leaving the MPR of the molecule under-analyzed and under-reported* ( Table 3). Considering the numerous functional motifs present in this region of the molecule, the high degree of regional polymorphism ( Table 1), and the potential effects of these polymorphisms on class II function, we encourage the HLA typing community to accelerate adoption of new protocols such as next-generation sequencing, which allow analysis of the *entire length* of each HLA molecule, and to include this important information is future database entries. This approach will provide information that could be used to divide existing HLA alleles into “sub-alleles” that may differ only in the MPR of the molecule, and could provide information crucial to determining the molecular mechanisms linking HLA class II to human disease. Two examples are discussed below.

**Table 3.** Frequency of reported full-length human leukocyte antigen class II sequences.

HLA chain	Total alleles ^a^	Number with membrane-proximal region sequence information ^b^	Fraction with reported membrane-proximal region sequence information
DRA	7	7	100%
DRB1	1,825	153	8%
DQA1	54	41	75%
DQB1	876	113	13%
DPA1	42	15	36%
DPB1	587	54	9%

^a^Total number of alleles for each locus listed in the IMGT/HLA database ( http://www.ebi.ac.uk/ipd/imgt/hla/) as of 4 Nov. 15.

^b^Number of alleles in IMGT/HLA database with sequence information for the membrane-proximal region of the molecule as of 4 Nov. 15. HLA, human leukocyte antigen.

Myasthenia gravis (MG) is an autoimmune disease with production of high-affinity antibodies to molecules of the neuromuscular junction (particularly the acetylcholine receptor). Generation of these antibodies requires HLA class II-restricted interactions between auto-reactive B cells and T cells. However, the molecular mechanisms underlying disease are not fully understood. Although a role for HLA class II in MG was appreciated in 1990, a series of articles between 2006 and 2012 established a crucial role for HLA-DQ in the disease ^57– 61^. Interestingly, many MG-associated HLA-DQ alleles bear multiple MPR polymorphisms when compared with the non-MG-associated “reference” alleles of DQA1*01:01:01 and DQB1*05:01:01 ( Table 1). These MPR polymorphisms are present in both chains and could impact class II-CD79 association, TM domain pairing/cholesterol binding, CT cAMP signaling, and CT ubiquitination. Because the precise molecular mechanism underlying the link between HLA-DQ and MG is still unclear, studies of MG-associated MPR polymorphisms and their effect on class II function may provide important new insights into the molecular mechanism of disease.

A relatively common variation in the HLA-DQ MPR is the presence of a CT proline-rich insert of eight amino acids (PQGPPPAG; Table 1) in approximately 10% of sequenced HLA-DQ β chains (8 out of 71). Interestingly, the first residue of this insert is the proline of the GP cAMP-signaling motif (see above). In alleles lacking the insert, the GP cAMP signaling motif is replaced by GL, which is unable to drive cAMP signaling. Because the cAMP signaling capability of the other HLA class II molecules is either untested (HLA-DR) or lacking (HLA-DP), it is possible that individuals bearing two DQ alleles lacking this insert and the resulting GP motif (likely about 1% of the population) could lack *all* HLA class II-driven cAMP signaling, which is known to be important for B-cell activation and antibody production. It would be interesting to see whether such individuals have a compromised humoral immune response.

### Open questions

• Are there HLA class II alleles that differ only in their MPR?
• What is the contribution of MPR polymorphisms in the molecular mechanisms linking HLA class II to human diseases?

## What lies ahead

MHC class II molecules mediate inter-cellular communications between class II-expressing APCs and CD4 T cells, meaning that peptide-class II complexes mediate *bidirectional* communications between these two populations of cells. The membrane-distal region of the class II molecule mediates peptide binding and TCR engagement, which functions to drive T-cell activation. However, the class II MPR impacts T-cell activation in multiple ways. By controlling class II ER retention, endocytic trafficking, and PLC entry, the MPR directly controls the class II peptidome available to drive T-cell activation. By controlling association with membrane sub-domains and the APC cytoskeleton, the MPR controls the “potency” of each peptide-class II complex. Future studies will need to elucidate how these MPR-driven functions are controlled for each and every peptide-class II complex and define their immunological impact. Answering the first part of the question will require the continued application of cutting-edge cellular and molecular approaches, as well as an appreciation of the molecular heterogeneity of the peptide-class II complexes under study. Answering the second part of the question will require extension into *in vivo* systems by using either genetically engineered experimental animals or samples from humans with MPR polymorphisms. Although results from some studies have been reported, such as a mouse expressing tail-less MHC class II molecules ^36^, these animals were tested under a limited set of experimental conditions. Future progress will require analysis of a greater number of mutations tested under a wider range of physiological conditions.

In addition to driving T-cell activation, class II molecules are signaling molecules and the MPR mediates *all* of these signaling events. Class II signaling is driven directly by the class II molecule (as in the case of cAMP signaling), by class II-associated signaling molecules such as CD79 and STING, or by membrane domains such as lipid rafts. Here, two pressing questions remain. First, given the heterogeneity of peptide-class II complexes, which peptide-class II complexes are capable of which forms of class II signaling and how is this regulated as a molecular level? Second, given the range of APCs, such as dendritic cells, B cells, and macrophages, what types of class II signaling are functional/important in which cells? Whereas some forms of signaling such as cAMP production may occur in many/all APCs, others such as CD79-driven signaling are more likely to be highly APC-specific. Here again, it will be critical to move to *in vivo* experimental systems to begin to unravel the immunological impact of these various class II pathways in relevant APC populations.

The function of the membrane- *distal* region of the class II molecule has been a long-term focus of immunologists. This emphasis has led to a detailed understanding of the biology and immunology of this molecular domain, which has provided the foundation for a deeper understanding of the role of class II in the human immune response. However, our understanding of the function of the class II MPR has lagged and future focus on this understudied topic holds much promise for significant strides. Future studies will not only increase our knowledge and appreciation of the immunological functions of this region of the molecule but will also form a foundation to understand how human polymorphisms impacting this region drive the molecular mechanisms of human disease. This next phase of investigation promises to be both exciting and enlightening.

## Abbreviations

αCP, alpha chain connecting peptide; APC, antigen-presenting cell; BCR, B-cell receptor; cAMP, cyclic AMP; CP, connecting peptide; CT, cytoplasmic tail; ER, endoplasmic reticulum; HLA, human leukocyte antigen; Ii, invariant chain (CD74); ITAM, immunoreceptor tyrosine-based activation motif; ITIM, immunoreceptor tyrosine-based inhibitory motif; mAb, monoclonal antibody; MG, myasthenia gravis; MHC, major histocompatibility complex; MPR, membrane-proximal region; PKC, protein kinase C; PLC, peptide-loading complex; STING, stimulator of interferon genes; TCR, T-cell receptor; TM, transmembrane.
